# Development of Novel Ceramic Microstructures from Sintered Biomass and Fly Ash Mixtures: Promoting Sustainability and Health

**DOI:** 10.3390/ma18071496

**Published:** 2025-03-27

**Authors:** Angeliki Moutsatsou, Eleni Katsika, Dimitrios Flegkas, Nikolaos Pagonis, Christina-Amalia Drosou, Aikaterini Itziou, Vayos Karayannis

**Affiliations:** 1 School of Chemical Engineering, National Technical University of Athens, 15773 Athens, Greece; angst@central.ntua.gr (A.M.); elenika23@gmail.com (E.K.); christine_dro@yahoo.gr (C.-A.D.); 2 Department of Chemical Engineering, School of Engineering, University of Western Macedonia, 50100 Kozani, Greece; flegkasdim@gmail.com (D.F.); nikpagn@gmail.com (N.P.); 3 School of Health Sciences, University of Western Macedonia, 50200 Ptolemaida, Greece; aitziou@uowm.gr

**Keywords:** novel ceramics, lignite fly ash, biomass, olive kernel ash, sintering, characterization, circular economy, health, sustainability

## Abstract

The valorization of agricultural and industrial solid by-products as secondary resources in the development of value-added materials can contribute to environmental health protection, particularly in the climate change era. Current advances in environmental legislation also encourage manufacturers to optimize waste management, upgrading and utilization towards resource conservation, energy efficiency and cost reduction in the context of a circular economy. In the present research, the elaboration of novel sustainable ceramics is investigated by sintering (at 800 °C for 2 or 6 h) of compacted mixtures composed of lignite fly ashes along with biomass ash (olive kernel ash) at different proportions. It appears that the chemical, mineralogical and morphological characteristics of these by-products promote their use as starting materials in ceramic engineering. Characterization and evaluation of the ceramics obtained via XRD and SEM-EDX analysis, as well as Vickers microhardness measurements, confirm the effectiveness of the consolidation process. In fact, the material derived from an 85% Class-C fly ash and 15% biomass ash compact, after 6 h sintering, exhibited greater results in terms of ceramic microstructure and microhardness (380 Hv), while a sintering time of 2 h was barely acceptable. The materials developed can be considered for use in various applications.

## 1. Introduction

The valorization of solid wastes, such as industrial by-products and agricultural residues, as secondary raw materials in the manufacturing of value-added products can contribute to environmental protection, resource conservation and cost reduction. Current advances in environmental legislation encourage manufacturers to optimize industrial by-product management and utilization [1,2]. Advancements in the production of added-value materials in an energy and cost-effective manner have been achieved by employing industrial and agricultural wastes. Fly ash (FA), bottom ash, iron blast-furnace slag, condensed silica fume, rice husk ash, soya husk ash, olive core ash, and municipal waste ash fall in the category of secondary or incidental products that derive from a chemical reaction or process, and although they are not as valuable as the main product, they are still capable of providing revenue. For instance, fly ash and biomass ash have been investigated for their utilization in cement and ceramic processing [3,4]. The presence of silica in agricultural and industrial wastes can be employed for the production of value-added products [5]. Advances in the sintering process are under investigation to enhance the efficiency of ceramic manufacturing [6]. Agricultural wastes have also been utilized for their conversion into activated carbons for adsorption applications [7].

Valorization of such waste was reported to have dual benefits: reduction of environmental impacts and production of sustainable materials. Several works have shown the value chain of olive biomass for energy, construction and other industries [8,9,10]. In addition, the most recent approaches, such as the transformation of wastes like eggshells into calcium phosphates for biomedical applications, represent an example of the partially discovered potential of wastes [11].

Various reports on the sustainability of bioenergy, among them the 2023 State of the Energy Union Report, cite that energy production from biomass in the EU-27 has grown strongly by 13% over the last decade. This growth has resulted from increased levels of power and heat production. Biomass accounted for about 15% of the EU’s total gross renewable electricity in 2021 and 6% of overall gross electricity production. In 2019, it also accounted for almost 19% of heat production and around 3% of total electricity generation in the EU [12].

Despite the potential, the exploitation of Greek resources for electric generation is underdeveloped, along with the weak management of residues that causes several environmental problems. In general, agriculture covers some 70% of the country’s area and underscores the high capacity for biomass resources, with Achaia being one of the leading regions rich in biomass residues. More specifically, its arable area is 190,754 acres, hosting 3,550,518 olive trees and producing about 77,580 metric tons of olive fruits yearly [12].

There are three main streams in olive oil production. The first stream consists of the product, olive oil, and the other two are the by-products. Here, the by-product streams are divided into two flows: (1) the solid waste, including olive kernel residuals, and (2) olive mill wastewater [13]. Olive kernel residuals have energetic capacity. In alignment with a circular economic model, olive kernel residuals are already used as a feedstock for energy production. The combustion of olive kernel waste to produce energy results in a by-product called olive kernel ash (OKA) [14]. The incorporation of OKA in a material enhances its hardness, endurance, elasticity and creep resistance while fatigue resistance and ductility are reduced. Additionally, the incorporation of OKA leads to increased viscosity in the material system and provides durability to higher temperatures [15,16]. OKA is applied in the construction sector, employed in materials like concrete, asphalt and bricks, and as activators of alkali materials or geopolymers [8,15,17]. Apart from civil engineering purposes, OKA bottom ash can be applied in plant development, as it contains nutrients that are important to the soil for plant development. The application of such a material into agriculture could reduce the utilization of commercial fertilizers [14]. However, the research regarding OKA and its potential applications is still limited.

Fly ash is a by-product of coal combustion for energy production [18]. According to ASTM 618 [19], FA is classified as Class F and Class C, based on the the sum of SiO_2_, Al_2_O_3_ and Fe_2_O_3_. If the sum is 70%, it is Class F, and if the sum is ≤70%, it is Class C. Additionally, the class determines the calcium composition. Class F is characterized by a low composition of calcium, while the reduced sum in Class C results in a higher composition of calcium. The production of Class F derives from the combustion of high-grade coals, such as anthracite and bituminous coals, while Class C derives from the combustion of low-grade coals, like lignite and sub-bituminous coal [18]. Pozzolanic reactivity and hydraulic behavior of FA strongly depend on its class. Moreover, a higher composition of amorphous silica results in greater pozzolanic reactivity [13].

In Greece, the major power plants are mainly situated in Northern Greece (Region of Western Macedonia), with one in Southern Greece (Megalopolis, Region of Peloponnese). The FA derived from Northern Greece has high Ca contents, resulting in Class C FA. On the other hand, the Southern Greece power plant produces siliceous ash (barely Class F).

FA consists of unburned carbon, magnetite, cenospheres, and solid fractions [20]. Even though there are plenty of sectors for the reutilization of FA, only 25% of the global production is reused [20,21]. Thus, a paradigm shift towards circularity and the reutilization of FA presents the most viable solution [22].

This valuable progress was made despite the fact that biomass residue and fly ash use in ceramic production is still a relatively unexplored frontier. Each of these wastes has shown promising chemical, mineralogical and morphological features concerning their application in ceramic engineering, though their application in this area has been limited. Most of the literature has centered on the use of biomass residues as by-products for energy production or as building materials [8,9,10], while fly ash is used in cement and concrete production [23,24]. Few investigators have researched the interaction between the two by-products when combined into sintered ceramic pieces, where their complementary properties could give rise to enhanced material performance [14,25].

The present study, therefore, attempts to fill this knowledge gap through a systematic exploration of the viability and advantages of olive kernel biomass and fly ash incorporation into ceramic production, underlining their transformational potential toward aligning industrial practices with sustainable goals. The examination will concentrate on olive kernel ash (OKA) and lignite fly ash (a highly calcareous one (FAAD) and a siliceous one (FAM)), major by-products of agro-industrial and energy production power stations, respectively. The high Ca can be expected to yield an interesting and possibly complex mineralogy in the sintered materials. Moreover, Ca may act as a flux, enabling melting to begin at lower temperatures (thus using less energy), which could provide beneficial formation of a thin liquid layer between ash particles during sintering, tending to pull them together. In this context, the elaboration of novel sustainable ceramics is investigated in the framework of a circular economy by sintering compacted mixtures composed of lignite fly ashes incorporated with biomass ash (OKA) at different proportions.

## 2. Materials and Methods

### 2.1. Raw Materials

One sample of highly calcareous ash (Class C), namely FAAD, and one classified as siliceous ash (marginally, Class F) FAM were collected from the electrostatic precipitators of the lignite-fired power stations of Greece. FAAD represents the fly ash derived from the power station of Agios Dimitrios in the Region of Western Macedonia (in Northern Greece), whereas FAM represents the fly ash that originated from the Megalopolis station (in Southern Greece). Of the total particles, 90% (D(v,0.9)) are smaller than 98.28 μm for FAAD and 199.87 μm for FAM. The chemical analysis results for these ashes are given in Table 1, which also includes the chemical analysis of OKA. The analysis was conducted by employing X-ray fluorescence (XRF (Houston, TX, USA), X-Lab 200).

### 2.2. Sample Preparation

The samples were prepared by mixing both types of FA along with OKA. FAM/OKA and FAAD/OKA samples were prepared with different compositions (5, 10 and 15% OKA). The mixtures were subjected to uniaxial cold pressing in a stainless steel die using a hydraulic press (Specac (Orpington, UK), 15011). The produced samples were disk-shaped compacts of 13 mm diameter and 3 mm width. The applied pressure was 700 MPa. Subsequently, the samples underwent sintering at 800 °C for 2 and 6 h in a laboratory chamber furnace (Thermconcept GmbH, Bremen, Germany, ΚL06/13). The sintered specimens were gradually cooled to ambient temperature in the furnace. Apart from the sintered specimens, green samples with the same proportions were prepared, which, after the pressing, did not participate in the sintering process and underwent instrumental analysis straight away. Table 2 illustrates a complete table, including ratios of FA and OKA, duration of sintering, temperature of sintering, applied pressure and surface area of the samples.

### 2.3. Instrumental Analysis

The initial samples of OKA, FAAD and FAM were analyzed by X-ray diffraction (XRD) to characterize their composition prior to sintering and compression. The XRD measurements were performed with a Siemens D5000 diffractometer (Siemens, Munich, Germany) (CU Ka, λ = 1.5406 Å) with a counting time of 5 s at each 0.05° interval. Furthermore, the particle size distribution of both FA classes was determined with a laser diffraction analyzer (Malvern Mastersizer Micro Ver. 2.19, Malvern Panalytical, Malvern, UK). OKA was examined for its microstructure and elemental composition through SEM (SEM—Jeol (Peabody, MA, USA), JSM-6400) and EDX analysis. Phase characterization of green and sintered specimens was determined by X-ray diffraction (XRD). The microstructures produced were studied using SEM and EDX analysis. Shrinkage of the samples was calculated as the volume change (%) upon sintering. Apparent density was measured according to the Archimedes principle using a specific apparatus (Shimadzu (Kyoto, Japan), SMK401-AUW220V). Vickers microhardness was measured with a load of 50 g and a dwell time of 15 s (Wilson Instruments (Campbellford, ON, Canada), Mode 402MVD, KNOOPIVICKERS TESTER). To enable reliable comparisons, mean microhardness values over five valid indentations per specimen were calculated.

## 3. Results and Discussion

**Figure 1:**
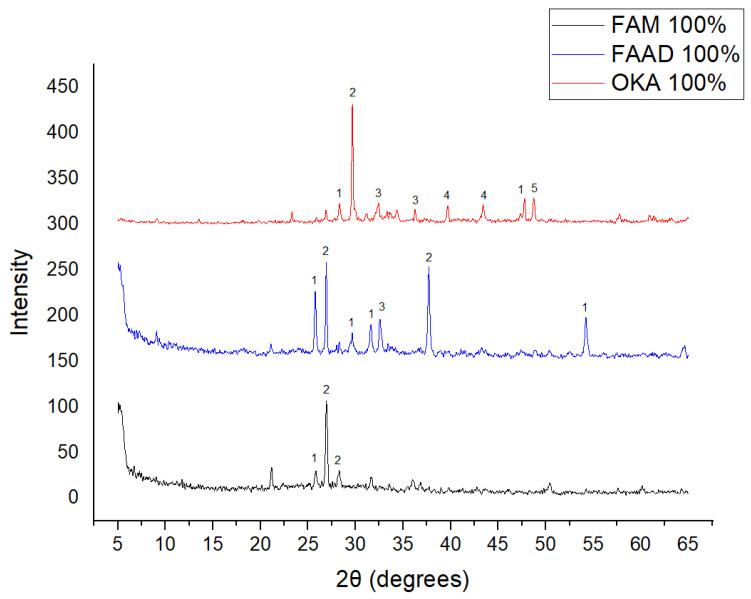
XRD analysis results of raw materials. The characterized peaks are: (1) potassium chloride, (2) quartz, (3) hematite, (4) calcite, (5) cristobalite.

The results of XRD analysis, for all ashes used as the raw materials, are shown in Figure 1. For OKA, key peaks indicate KCl (28.32°), quartz (29.68°), hematite (32.42°, 36.22°), calcite (39.74°, 43.44°) and cristobalite (48.78°). In FAAD, anhydrite appears at 25.8°, 29.6°, 31.56° and 54.1°, with quartz (26.9°, 37.7°) and hematite (32.5°) also present. FAM shows dominant quartz (26.9°, 28.3°), mullite (25.8°) and an ambiguous peak at 21.1°. For 85%FAM15%OKA, quartz (26.8°), calcite (29.6°) and cristobalite (25.8°) were identified, while 90%FAM10%OKA exhibits quartz (21.2°, 26.9°), mullite (25.7°) and cristobalite (28.1°). 95%FAM5%OKA contains quartz (20.9°, 26.7°), arcanite/sylvite (25.6°, 28.1°) and calcite (35.8°). After sintering for 2 h, 85%FAM15%OKA presents quartz (21.2°, 27.08°), hielscherite (25.8°, 31.8°) and maghemite (50.5°), while 90%FAM10%OKA shows anhydrite (21°), quartz (25.6°, 26.8°), gehlenite (31.4°), hematite (35.7°) and mullite (50.3°). Lastly, 95%FAM5%OKA features quartz (21.2°, 26.9°, 36.08°), anhydrite (25.9°) and sylvite (28.1°). All results were cross-referenced with the COD database.

According to the laser diffraction analyzer, the fraction of FAAD under 30.92 μm is 50% and 90% under 98.28 μm, whereas, for FAM, only 50% of its granules are under 98.92 μm and 90% under 199.87 μm. These results indicate that Class C FA behaves better than Class F. This is attributed to the fact that smaller granules result in higher quality material due to their increased surface area, better uniformity, smoother compaction and enhanced mechanical properties [26,27].

**Figure 2:**
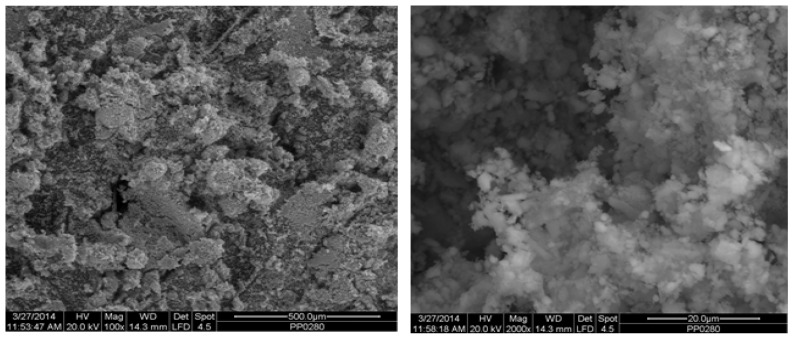
SEM analysis of the green OKA: at ×100 magnification with a 500 μm scale bar (**left**) and at ×2000 magnification with a 20 μm scale bar (**right**).

**Figure 3:**
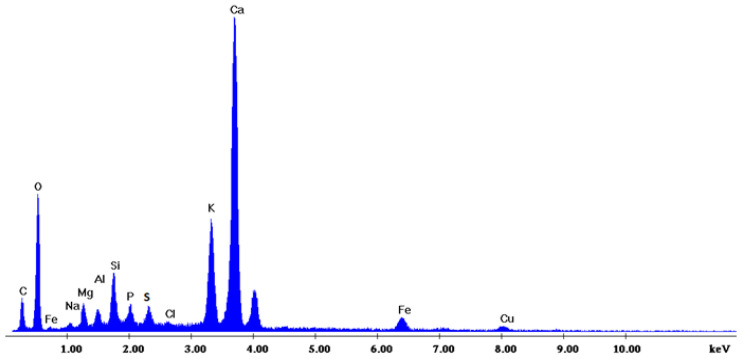
EDX analysis of the OKA.

Figure 2 depicts the internal microstructure of OKA in different scale magnifications. It is observed that particles agglomerate, and their surface seems rough and uneven, indicating high surface area material. Figure 3 illustrates the elemental composition. Plenty of elements appear, including Ca and O, in the highest compositions. Table 3 demonstrates the elemental composition of EDAX analysis.

**Figure 4:**
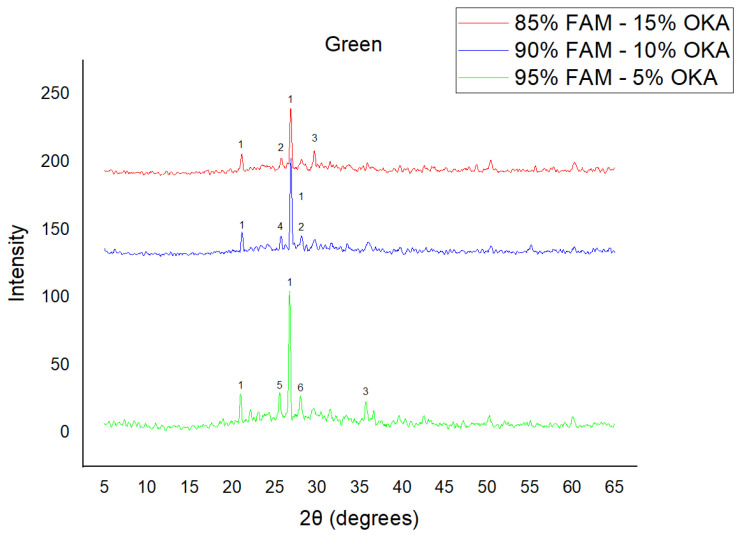
XRD analysis of green FAM/OKA mixture in different compositions (15, 10 and 5% OKA). The characteristic peaks are (1) quartz, (2) cristoballite, (3) calcite, (4) mullite, (5) arcanite, (6) sylvite.

**Figure 5:**
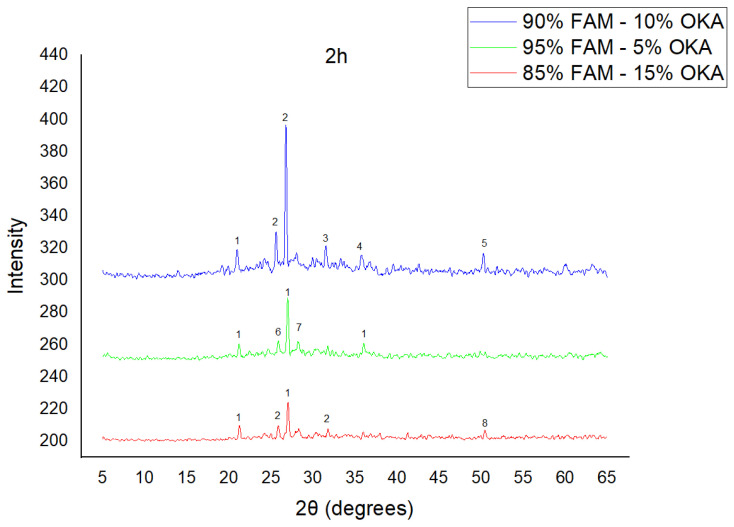
XRD analysis of FAM/OKA mixture in different compositions (15, 10 and 5% OKA) and sintered at 800 °C for 2 h. The corresponding peaks are (1) quartz, (2) hielscherite, (3) gehlenite, (4) hematite, (5) mullite, (6) anhydrite, (7) sylvite, (8) maghemite.

**Figure 6:**
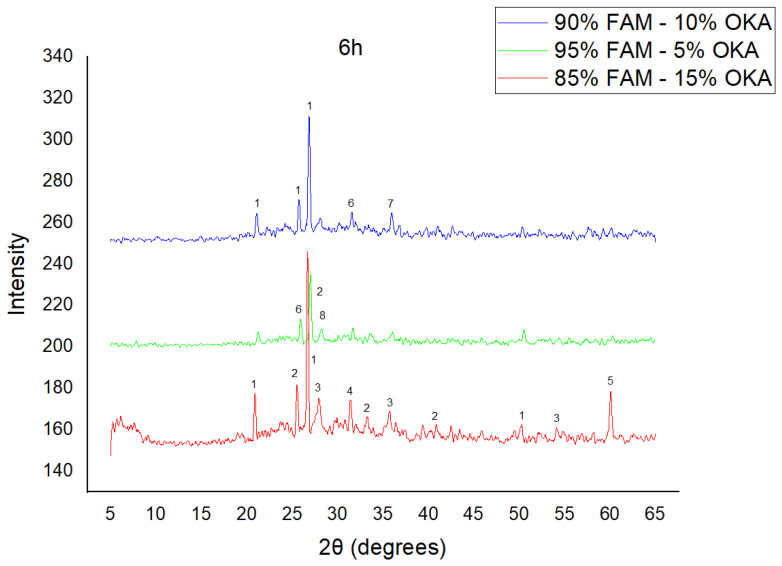
XRD analysis of FAM/OKA mixture in different compositions (15, 10 and 5% OKA) at 6 h sintering time for 800 °C. The corresponding peaks are (1) quartz, (2) mullite, (3) rutile, (4) gehlenite, (5) periclase, (6) anhydrite, (7) maghemite, (8) sylvite.

**Figure 7:**
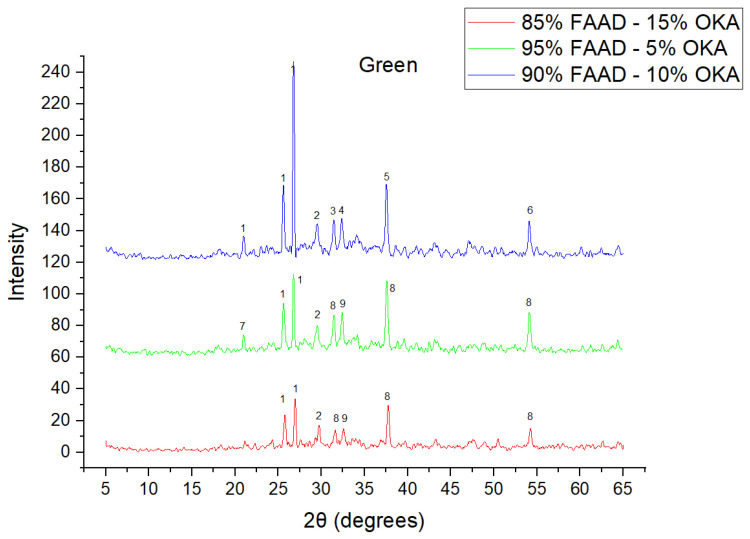
XRD analysis of green FAAD/OKA mixture in different compositions (15, 10 and 5% OKA). The corresponding peaks are (1) quartz, (2) calcite, (3) hematite, (4) maghemite, (5) periclase, (6) rutile, (7) goethite, (8) lime, (9) sodalite.

**Figure 8:**
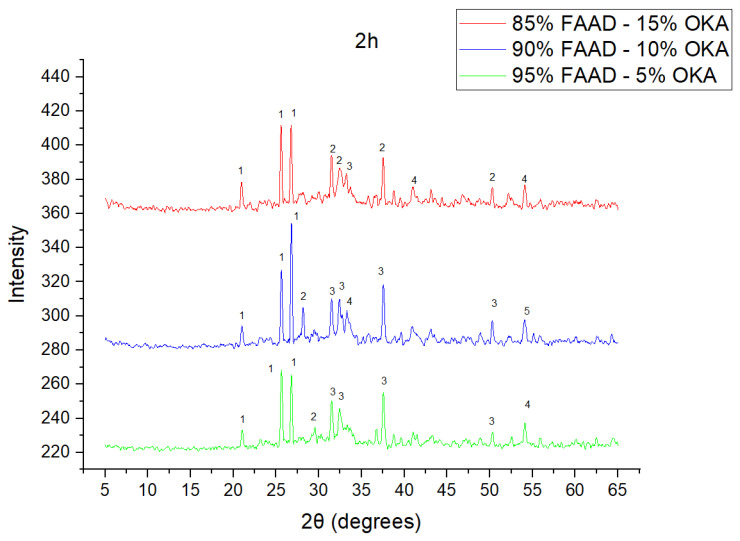
XRD analysis of FAAD/OKA mixture in different compositions (15, 10 and 5% OKA) and sintered at 800 °C for 2 h. The corresponding peaks are (1) quartz, (2) calcite, (3) mullite, (4) maghemite, (5) hematite.

**Figure 9:**
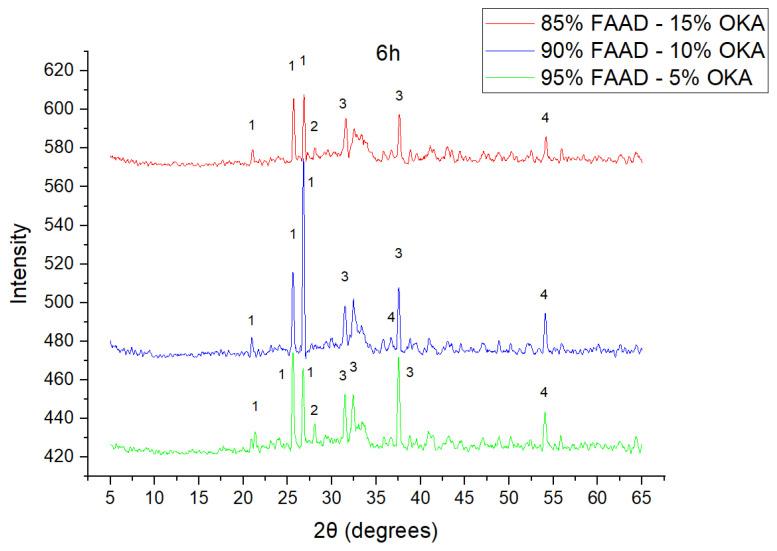
XRD analysis of FAAD/OKA mixture in different compositions (15, 10 and 5% OKA) and sintered at 800 °C for 6 h. The corresponding peaks are (1) quartz, (2) calcite, (3) mullite, (4) hematite.

As shown in Figure 4, Figure 5 and Figure 6, there is a distinct crystalline phase influenced by higher sintering time. In the 2 h specimens, dominant peaks correspond to quartz, while additional peaks suggest the strong presence of anhydrite, feldspar and hematite. In the 6 h sintered specimens, other phases, such as mullite and cristoballite, emerge stronger, which confirms that they are more distinct with longer sintering. These results highlight the ongoing crystallization during sintering, which is influenced by the composition and sintering duration. Regarding the FAAD/OKA specimens, as shown in Figure 7, Figure 8 and Figure 9, it is visible that quartz is dominant in both the 2 h and 6 h sintered samples, while mullite and hematite formation is enhanced with further sintering. The extent of crystallization highly depends on the composition and the sintering duration.

**Figure 10:**
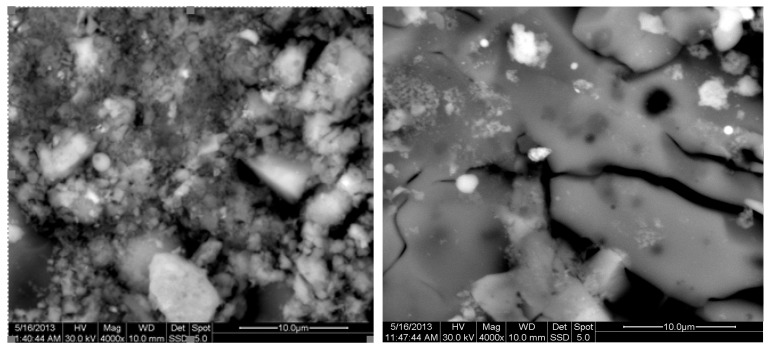
SEM analysis of 90%FAM10%OKA mixture: green at ×4000 magnification with a 10 μm scale bar (**left**) and sintered at 800 °C for 2 h at ×4000 magnification with a 10 μm scale bar (**right**).

**Figure 11:**
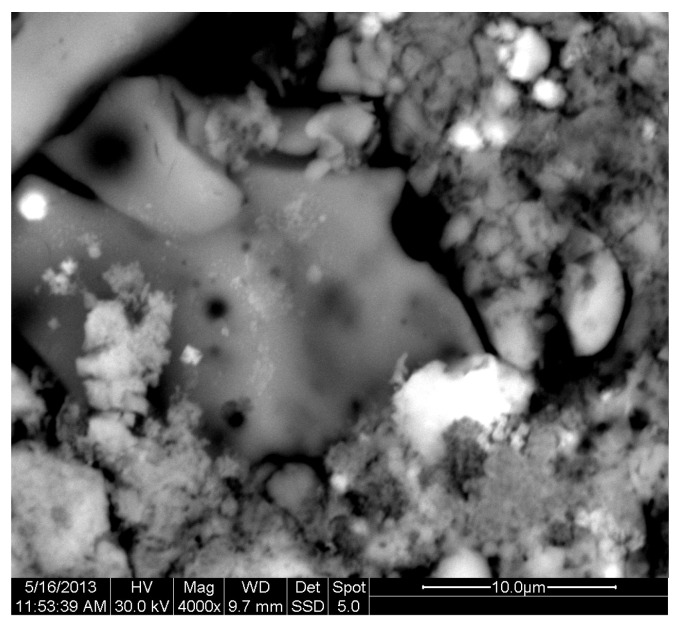
SEM analysis of 90%FAM10%OKA mixture: sintered at 800 °C for 6 h at ×4000 magnification with a 10 μm scale bar.

**Figure 12:**
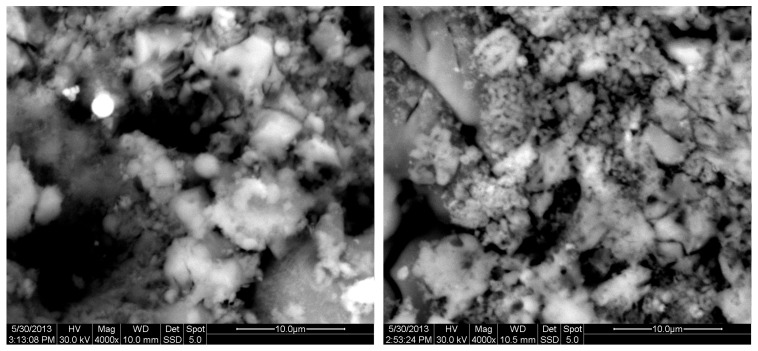
SEM analysis of 85%FAM15%OKA mixture: green at ×4000 magnification with a 10 μm scale bar (**left**) and sintered at 800 °C for 2 h at ×4000 magnification with a 10 μm scale bar (**right**).

**Figure 13:**
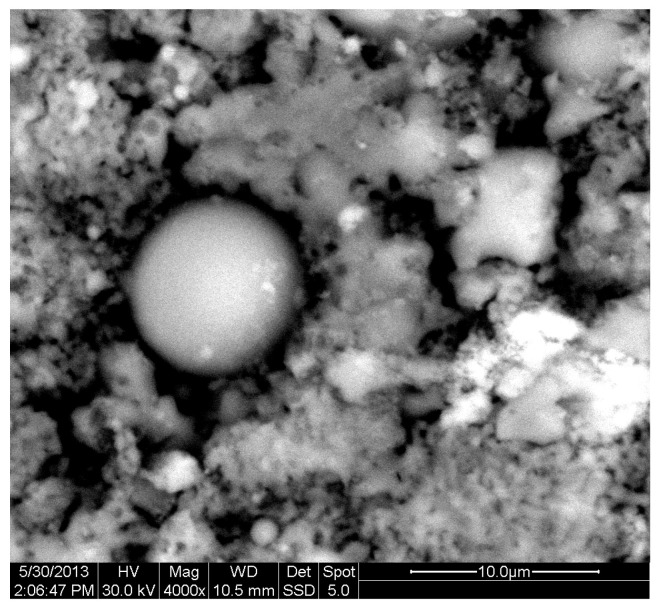
SEM analysis of 85%FAM15%OKA mixture: sintered at 800 °C for 6 h at ×4000 magnification with a 10 μm scale bar.

**Figure 14:**
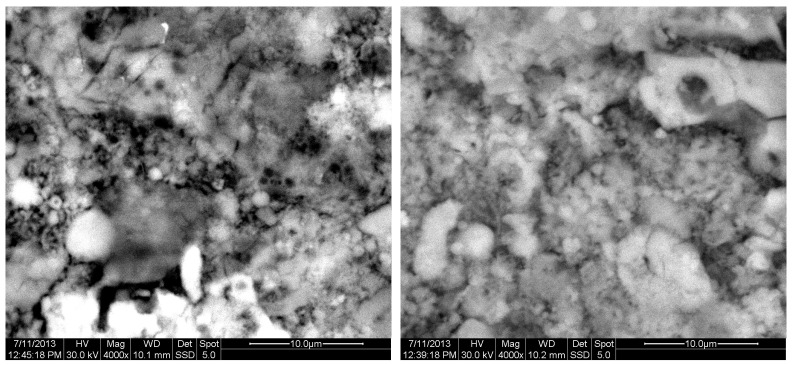
SEM analysis of 85%FAAD15%OKA mixture: green at ×4000 magnification with a 10 μm scale bar (**left**) and sintered at 800 °C for 6 h at ×4000 magnification with a 10 μm scale bar (**right**).

**Figure 15:**
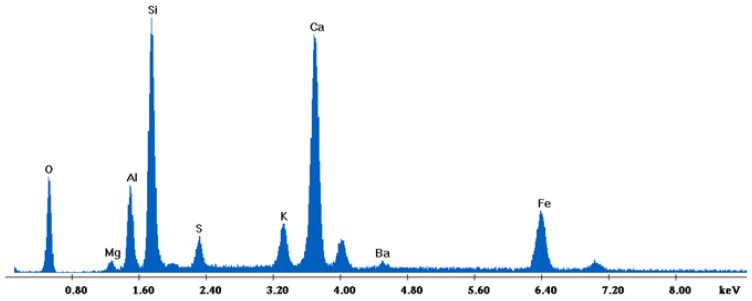
EDX analysis of 85%FAM15%OKA sintered at 800 °C for 6 h.

**Figure 16:**
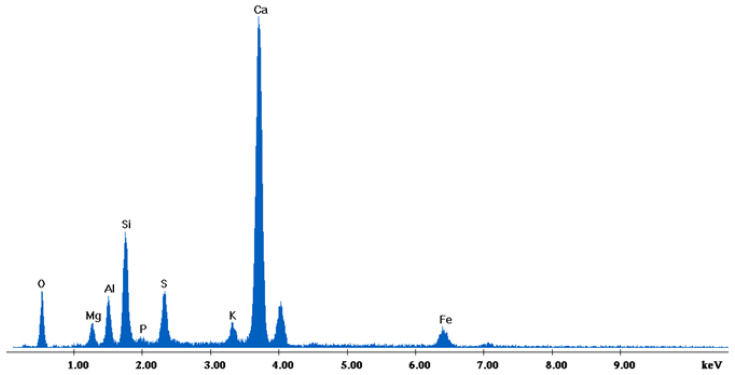
EDX analysis of 85%FAAD15%OKA sintered at 800 °C for 6 h.

The results of the consolidation process can be evaluated upon microstructural observation of sintered specimens via SEM analyses (see Figure 10, Figure 11, Figure 12, Figure 13, Figure 14 and Figure 15). From Figure 10, when using the 2 h heating time, a reasonably sintered and rather rough matrix is shown where quartz is located [28]. On the other hand, a finer microstructure is obtained when the 6 h heating time is employed (Figure 10 and Figure 11). A continuous network of characteristic solid-state sintering necks can be clearly seen. According to Figure 10, Figure 11 and Figure 12, the laser diffraction analyzer analysis is confirmed because the particles involved in the FAAD mixtures are finer than those in FAM. Also, because of the fly ash, hollow particles (cenospheres) can be distinguished (see Figure 13). The best structure is presented for the 6 h heating time and the 85%FAAD/15%OKA mixture. The shrinkage of the sintered specimens is in the range of 1–5%. The density lies in the range of 3.1–6.0 g·cm^−3^. Figure 15 and Figure 16 illustrate the EDAX results for 85%FAM15%OKA and 85%FAAD15%OKA, respectively. Comparing these figures, it is obvious that the incorporation of more OKA elevates the composition of Ca in the mixture, both in FAM and FAAD-based materials. Table 4 and Table 5 represent the elemental composition of EDAX analysis. In the FAM-based material, Ca composition is enhanced, and it is relatively lower than Si (18.96% Ca and 20.18% Si). On the other hand, in FAAD-based material, the Ca composition is almost three times higher than Si (33.55% Ca and 11.94% Si).

**Figure 17:**
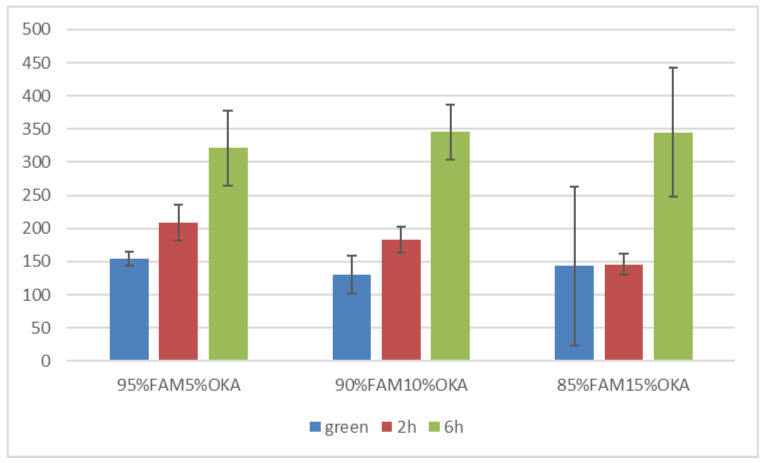
Vickers microhardness of FAM/OKA mixtures in different compositions and different sintering times (green, 2 h, and 6 h).

**Figure 18:**
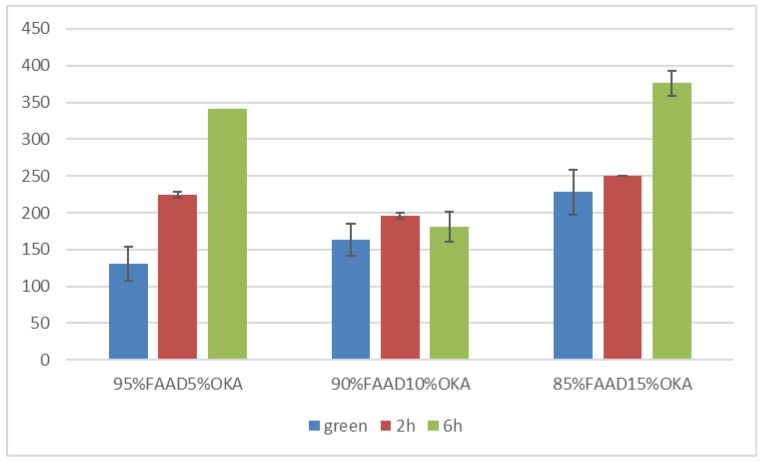
Vickers microhardness of FAAD/OKA mixtures in different compositions and different sintering times (green, 2 h, and 6 h).

Vickers microhardness is presented in Figure 17 and Figure 18. It is obvious that in all cases, the green specimen exhibited lower microhardness values. After sintering, the specimens showed elevated microhardness values. Subsequently, samples sintered for 6 h were harder than those of 2 h, with one exception in the FAAD case, where 90%FAAD/10% OKA after 6 h of sintering was less hard than the 2 h specimen.

Another important observation is that the incorporation of OKA into green FAM-based materials results in degraded hardness. As depicted in Figure 17, green 95%FAM5%OK is harder than both green 90%FAM10%OKA and green 85%FAM15%OKA. The 2 h sintering seems to have a greater effect on the 95%FAM5%OKA specimen, enhancing its microhardness more than the other two specimens. The 6 h sintering enhanced the microhardness of the FAM/OKA materials even more. As a result, the duration of sintering was effective for specimens with elevated OKA composition. On the other hand, in Figure 18, the FAAD-based materials exhibited greater microhardness values with more OKA composition in green samples. Sintering enhanced hardness, with the exception of 90%FAAD10%OKA, as previously mentioned, where the microhardness was lower after 6 h of sintering compared to 2 h. The greatest microhardness values were observed for 85%FAAD15%OKA after 6 h of sintering.

A novel ceramic material was fabricated by using industrial wastes as feedstock. In this case, FA powder of the discriminated class was mixed with OKA in different compositions. The produced materials were characterized by their crystallography, microstructure, elemental composition, and microhardness. Both FAM and FAAD exhibited excellent microstructural and mechanical properties. However, it was indicated that the FA class had an effect on the final material properties. According to SEM and Vickers microhardness analysis, Class C-based materials demonstrated superior properties. More specifically, 85%FAAD15%OKA with a 6 h sintering time exhibited the best results, as it possessed better microstructure and a larger surface area, attributed to the small granules of FAAD powder and better microhardness.

Based on microstructure and microhardness analyses, it was revealed that the sintering duration had an impact on the final material produced. Regarding microstructure, elevated sintering time led to materials with a continuous structural network. In the particular study, sintering of 6 h provided a sufficient amount of time for the neck formation of the granules, in contrast to the sintering of 2 h, which was also effective but in a reduced manner. More time for sintering resulted in higher hardness. Both FAM and FAAD-based materials exhibited elevated microhardness values when they were exposed to higher sintering time, with only the exception of 90%FAAD10%OKA, where 2 h of sintering resulted in reverse results. The incorporation of OKA into fly ash mixtures, combined with sintering time, also enhanced the hardness of the final material. The materials with a higher OKA composition and sintering time demonstrated higher microhardness values, with the exception of 90%FAAD10%OKA, as mentioned above.

As aforementioned, both OKA and FA are traditionally used in the construction sector, for example, in concrete, bricks and asphalt. The combination and sintering of both materials appear to be capable of forming an innovative construction ceramic material. Class C FA, along with OKA, present a particular interest because of their elevated composition in CaO [29]. Another important field of application may be in catalytic systems for advanced wastewater treatment. The composition of many oxides, and particularly the high ratio in aluminosilicates, make the ceramics produced a potential candidate for support applications, certainly after further optimization and testing [30]. Also, this should be accompanied by photocatalytic activity studies for the degradation of pollutants in wastewater and air and self-cleaning processes, as FA-based photocatalysts have been reported to be efficient for such types of applications [31,32]. However, further investigation is required to evaluate the performance of the ceramic materials produced. In addition, an innovative approach can be the examination of such wastes and the ceramics developed in the current work in the biomedical sector as scaffolds in bone regeneration, also due to the oxide composition. However, biocompatibility has been proven to be low, and osteoconductive properties have also been degraded so far [33]. Despite that, research for the employment of FA particles is ongoing, especially regarding cenospheres, which have proven to be both biocompatible and bioactive [34]. For this purpose, a comprehensive assessment must be carried out to ascertain their possible toxicity. Evaluating cytotoxicity, histotoxicity and genotoxicity has become essential [35,36]. ISO and FDA offer protocols and guidelines that can be utilized for this purpose, endorsing the safety of newly produced materials when they demonstrate cytocompatibility in vitro. However, in vitro assessments cannot be directly connected to the results in the whole organism, and therefore, a thorough clinical evaluation of the potential biomaterials must be carried out, addressing factors like oxidative stress and inflammation. A diverse range of evaluations is essential to fully grasp the characteristics of the material’s toxicity, ensuring human health and safety [37].

This study constitutes the base material for further research due to its potential in various fields. Further mechanical testing should be conducted, such as stress–strain and fatigue tests, to ensure the material’s durability, as well as thermal testing to determine the material’s behavior in various thermal conditions. Variations in the fabrication process are also proposed by increasing the firing temperature. Additional sintering processes could include flash sintering, micro-wave sintering and many other sustainable processes to achieve faster sintering, eco-friendliness and reductions in production costs. Moreover, biocompatibility tests, including cell growth, proliferation and cytotoxicity, should be conducted to evaluate its potential in the biomedical sector.

## 4. Conclusions

This research underlines the viability of the secondary resources investigated for the production of eco-ceramics as a contribution to resource conservation and waste valorization in line with the principles of the circular economy, environmental sustainability and health. The produced materials had satisfactory microhardness and microstructure results, which are encouraging for further research to optimize, tailor and model the material properties. The fabrication of a material derived from FA and OKA seems viable and could enhance sustainability and eco-friendliness. In addition, the utilization of such wastes reduces production costs.

Further optimization of the manufacturing process should be evaluated by altering the sintering process to a more enhanced and viable one, like hot pressing, flash sintering or microwave sintering. Such a modification could allow for increases in the firing temperature without burdening the environment and without increasing energy demands. Moreover, these sintering processes would reduce the duration of sintering, as both are more rapid than traditional methods, as the densification process is accelerated, resulting in enhanced mechanical properties [38,39,40].

Both of the ashes are utilized in the industry sector. Thus, the combination of these two materials could lead to a material with interesting properties in such a field. Other potential application sectors include construction materials, catalytic/photocatalytic systems and biomedical engineering. These sectors demand appropriate tests of microstructure, mechanical and surface properties. In addition, biomedicine demands further biocompatibility tests to ensure the safety of the material.

In conclusion, the ceramics obtained exhibited interesting microstructure and hardness values. This encouraging result could be the driving force to further evaluate the material’s mechanical properties and enhance them. Moreover, research at higher sintering temperatures and extended characterizations of processed ashes will enable this approach to be thoroughly optimized and even modeled for specific applications.

## Figures and Tables

**Table 1:** Chemical analysis of the ashes.

Component	SiO_2_	Al_2_O_3_	Fe_2_O_3_	CaO	MgO	K_2_O	Na_2_O	SO_3_	TiO_2_
FAAD (%)	30.16	14.93	5.1	34.99	2.69	0.4	1.01	6.28	-
FAM (%)	49.54	19.25	8.44	11.82	2.27	1.81	0.53	3.91	-
OKA (%)	32.6	3.1	1.9	10.2	3.8	27.2	4.2	5.0	0.1

**Table 2:** A complete presentation of all samples, including FA and OKA content and manufacturing conditions.

	FA Content (%wt)	OKA Content (%wt)	Sintering Duration (h)	Sintering Temperature (°C)	Applied Pressure (ΜPa)	Diameter (mm^2^)
95%FAAD5%OKA	95	5	2	800	700	13
95%FAAD5%OKA	95	5	6	800	700	13
95%FAM5%OKA	95	5	2	800	700	13
95%FAM5%OKA	95	5	6	800	700	13
90%FAAD10%OKA	90	10	2	800	700	13
90%FAAD10%OKA	90	10	6	800	700	13
90%FAM10%OKA	90	10	2	800	700	13
90%FAM10%OKA	90	10	6	800	700	13
85%FAAD15%OKA	85	15	2	800	700	13
85%FAAD15%OKA	85	15	6	800	700	13
85%FAM15%OKA	85	15	2	800	700	13
85%FAM15%OKA	85	15	6	800	700	13
Green 95%FA5%OKA	95	15	-	-	700	13
Green 90%FA10%OKA	90	10	-	-	700	13
Green 85%FA15%OKA	85	15	-	-	700	13

**Table 3:** Table based on the elemental composition provided by EDX analysis of OKA. They are shown in order of weight percentage of the element (Wt%), atomic percentage of the element (At%), the ratio of X-ray intensity of the element (K-ratio) and the correction factors used in EDX quantification (Z, A, F).

Element	Wt%	At%	K-Ratio	Z	A	F
C	6.75	12.78	0.0198	1.0591	0.2761	1.0007
O	39.10	55.54	0.0618	1.0412	0.1518	1.0002
Na	0.70	0.70	0.0021	0.9743	0.3055	1.0014
Mg	2.00	1.87	0.0087	0.9987	0.4360	1.0023
Al	1.17	0.98	0.0064	0.9692	0.5649	1.0041
Si	3.42	2.77	0.0235	0.9974	0.6860	1.0058
P	1.42	1.04	0.0105	0.9635	0.7618	1.0094
Cl	0.24	0.16	0.0021	0.9428	0.8843	1.0279
K	8.52	4.95	0.0814	0.9480	0.9574	1.0534
Ca	30.11	17.07	0.2745	0.9701	0.9385	1.0016
Fe	3.20	1.30	0.0275	0.8826	0.9679	1.0039
Cu	1.95	0.70	0.0164	0.8530	0.9867	1.0015
S	1.41	0.16	0.0091	0.6266	1.0226	1.0000

**Table 4:** Table based on the elemental composition provided by EDX analysis of 85%FAM15%OKA sintered at 800 °C for 6 h. They are shown in the order of weight percentage of the element (Wt%), atomic percentage of the element (At%), the ratio of X-ray intensity of the element (K-ratio) and the correction factors used in EDX quantification (Z, A, F).

Element	Wt%	At%	K-Ratio	Z	A	F
O	37.09	56.27	0.0615	1.0400	0.1594	1.0005
Mg	1.06	1.05	0.0032	1.0024	0.3028	1.0068
Al	6.40	5.76	0.0260	0.9739	0.4134	1.0099
Si	20.18	17.44	0.0978	1.0033	0.4808	1.0044
S	2.25	1.70	0.0122	0.9969	0.5401	1.0102
K	3.29	2.05	0.0263	0.9554	0.8068	1.0367
Ca	18.96	11.48	0.1568	0.9775	0.8408	1.0065
Ba	1.69	0.30	0.0129	0.7714	0.9802	1.0107
Fe	9.08	3.95	0.0773	0.9022	0.9426	1.0000

**Table 5:** Table based on the elemental composition provided by EDX analysis of 85%FAAD15%OKA sintered at 800 °C for 6 h. They are shown in order of weight percentage of the element (Wt%), atomic percentage of the element (At%), the ratio of X-ray intensity of the element (K-ratio) and the correction factors used in EDX quantification (Z, A, F).

Element	Wt%	At%	K-Ratio	Z	A	F
O	34.30	53.07	0.0433	1.0356	0.1219	1.0004
Mg	3.51	3.58	0.0112	0.9982	0.3170	1.0059
Al	4.82	4.42	0.0194	0.9698	0.4116	1.0089
Si	11.94	10.53	0.0597	0.9990	0.4961	1.0081
P	1.04	0.83	0.0051	0.9668	0.4995	1.0127
S	5.04	3.89	0.0310	0.9927	0.6093	1.0164
K	2.06	1.30	0.0174	0.9512	0.8335	1.0643
Ca	33.55	20.72	0.2855	0.9732	0.8725	1.0019
Fe	3.73	1.66	0.0309	0.8983	0.9200	1.0000

## Data Availability

The original contributions presented in this study are included in the article. Further inquiries can be directed to the corresponding author.

## Abbreviations

The following abbreviations are used in this manuscript:

OKA	Olive kernel biomass ash
FAAD	Highly-calcareous fly ash
FAM	Siliceous fly ash
XRD	X-ray diffraction
SEM	Scanning electron microscopy
EDAX	Energy-dispersive X-ray spectroscopy
